# P05 Osteoporosis in thalassemia; a case with multifactorial aetiology

**DOI:** 10.1093/rap/rkad070.026

**Published:** 2023-09-27

**Authors:** Chamith Rosa, Madhuri Shivaraj, Kaushik Chaudhuri

**Affiliations:** University Hospitals Coventry and Warwickshire NHS Trust, Coventry, United Kingdom; University Hospitals Coventry and Warwickshire NHS Trust, Coventry, United Kingdom; University Hospitals Coventry and Warwickshire NHS Trust, Coventry, United Kingdom

## Abstract

**Introduction:**

Thalassemia patients are prone to osteoporosis due to multifactorial reasons, including anaemia, bone marrow expansion, iron overload and hypogonadism.

**Case description:**

A 41-year-old British Asian male with beta thalassemia major was referred to rheumatology in 2018 for osteoporosis. In 2008, aged 27 years, whilst playing football, he developed a distal radial fracture and ulnar styloid process fracture and a DEXA scan had revealed low bone mineral density (BMD) for age. Subsequently, he was started on Ibandronic acid by his haematologist for a total of 10 years. After he was referred to rheumatology in 2018, bisphosphonates were discontinued as he had 5% improvement in BMD, a decade of bisphosphonates and no further fractures.

Further assessment revealed multiple contributory factors for his osteoporosis.

• Consistency low vitamin D – 9.6 nmol/L in 2009, average value of 20

• He was diagnosed with hypogonadism, poor compliance with IM testosterone injections

• He had diabetes which was not optimally controlled

• Evidence of iron overload with average serum ferritin of 2000 ug/L with deferasirox

While these factors were optimised, it was decided to adopt a watch and wait policy with regards to specific osteoporosis treatment.

Review in 2023 revealed serum testosterone level of 20.1 nmol/L (6.9-23.2) with better compliance with testosterone injections. Fructosamine levels were 310 (normal range 200-285) but it has reduced from a peak of 370. Serum ferritin levels have reduced to 776 down from a high of 5600. Normal vitamin D levels were maintained.

The DEXA scan in 2023 revealed a spine T score of −2.4 which showed 10.2% increase in bone mineral density. DEXA scan in 2020 revealed T score of −3.1 in spine and −1.9 in hips. The P1NP level was 56 (19-69) which has reduced from 100 in 2021. Improvements in BMD and bone turnover markers were achieved through optimising contributory factors without any specific osteoporosis treatment. He will be monitored with periodic BMD and bone turnover markers.

**Discussion:**

Anaemia and subsequent bone marrow expansion is a recognised cause for osteoporosis in thalassemia patients. While regular blood transfusions inhibit bone marrow expansion, ineffective erythropoiesis is not fully supressed. This patient received 3 units of blood every 4 weeks. Bone marrow expansion occurs mostly in trabecular bone resulting in low BMD is areas which generally have increased amount of trabecular bone such as the spine. Our patient had a lower BMD in the spine compared to the femoral neck.

Iron overload directly disrupts bone formation and can result in endocrinopathies which contributes to osteoporosis. Hypogonadism is another well-recognised cause and is primarily results from iron deposition in pituitary and at times in the gonads. Hormonal replacement therapy reduces bone loss. Studies have shown that hypogonadic patients have more severe osteoporosis and predominant involvement of the femoral neck.

Iron overload can cause diabetes mellitus and impaired growth hormone/ insulin growth factor-1 (IGF-1) axis which can contribute to osteoporosis. Our patient did have diabetes but had normal IGF-1 levels when checked previously.

Deferoxamine which is one of iron chelating agents have been shown to reduce collagen synthesis and increases osteoblast apoptosis which might contribute to the development of osteoporosis.

Vitamin D levels are well known to be low in patients with thalassemia compared to the general population.

While there are no RCT’s to show that bisphosphonates reduce fracture risk, surrogate markers such as increased BMD and reduction in bone turnover markers are known to occur. Therefore, current guidance (Standards for the Clinical Care of Children and Adults with Thalassaemia in the UK) recommends bisphosphonates to be considered in patients with a low BMD for age (Z-score < −2.0, T-score < −2.5 if post-menopausal or over 50), if there are fragility fractures or falling BMD despite adequate vitamin D levels, and hormone supplementation.

**Key learning points:**

Learning point

Important to assess for osteoporosis in thalassaemic patients.

There are multiple factors contributing to the development of osteoporosis which needs to be addressed together with endocrinologist and haematologist.

Bisphosphonates are recommended though further RCT evidence is required to support this

